# Antimicrobial effects of different concentrations of simvastatin versus triple antibiotic paste on *Enterococcus faecalis* biofilms at different stages of development

**DOI:** 10.34172/joddd.2022.026

**Published:** 2022-11-15

**Authors:** Saeed Rahimi, Negin Ghasemi, Paria Davoudi, Isun Taleb, Mehran Farajollahi, Naghmeh Rahimi Darehchi, Ezatolah Kazeminejad

**Affiliations:** ^1^Dental and Periodontal Research Center, Department of Endodontics, Faculty of Dentistry, Tabriz University of Medical Sciences, Tabriz, Iran; ^2^Department of Endodontics, Faculty of Dentistry, Tabriz University of Medical Sciences, Tabriz, Iran; ^3^Department of Endodontics, Faculty of Dentistry, Shahid Beheshti Medical University, Tehran, Iran; ^4^Private Practice, Tabriz, Iran; ^5^Dental Student, Semmelweis Dental School, Budapest, Hungary; ^6^Dental Research Center, Golestan University of Medical Sciences, Grogan, Iran

**Keywords:** Enterococcus faecalis, Antibacterial agents, Simvastatin, Root canal preparation

## Abstract

**Background.** This study assessed the antimicrobial effects of different concentrations of simvastatin versus triple antibiotic paste (TAP) on *Enterococcus faecalis* biofilms at different stages of development.

**Methods.** In this in vitro study, 70 human single-rooted mature premolars were decoronated, instrumented, and autoclave-sterilized. Next, an *E. faecalis* suspension was prepared and inoculated into the canals to obtain 4- and 6-week biofilms. After ensuring biofilm formation, the samples in each group were randomly assigned to 5 subgroups (n=12): 1 mg/mL TAP, 10 mg/ mL TAP, 1 mg/mL simvastatin, 10 mg/mL simvastatin, and positive control (phosphate-buffered saline solution). The medicaments were applied in the canals, and the teeth were incubated for one week. Dentin samples were collected by a rotary file, cultured, and the number of *E. faecalis* colonies was counted. The Kruskal-Wallis, Mann-Whitney U, and Wilcoxon tests were used for data analysis (α=0.05).

**Results.** There were significant differences in colony counts between the two concentrations of TAP and the control group against both 4- and 6-week biofilms (*P*<0.05). The antibacterial effect of 10 mg/mL TAP and simvastatin was stronger than that of 1 mg/mL concentration against the 4- and 6-week *E. faecalis* biofilms (*P*<0.05). Furthermore, 10 mg/mL TAP and simvastatin were more effective against the 4-week biofilms than the 6-week biofilms (*P*<0.05).

**Conclusion.** According to the present results and since biofilms may remain viable in the root canal system for weeks to months, applying 10 mg/mL TAP and simvastatin might be more effective.

## Introduction

 Successful endodontic treatment and a predictable outcome can only be achieved by effective root canal debridement, irrigation, and elimination of microorganisms.^1,2^ Intracanal medicaments are used to enhance root canal disinfection.^3^ In regenerative endodontic procedures, minimal mechanical root canal preparation is required, and biomaterials are applied to induce odontogenic differentiation of dental pulp stem cells (DPSCs) ^4^ and enable clinical maturation of the root.^5,6^

 As a Gram-positive, facultative anaerobe, *Enterococcus faecalis *can survive root canal treatment.^7^ It can form biofilms and survive prolonged periods of nutritional deprivation. It can also resist acidic and basic conditions and is hard to eradicate.^8^ *E. faecalis* is responsible for 20‒70% of endodontic failures and secondary endodontic infections.^9^

 Triple antibiotic paste (TAP), a mixture of minocycline, ciprofloxacin, and metronidazole, is commonly used as an intracanal medicament during regenerative endodontic therapy.^10^ High concentrations of TAP (>10 mg/mL) can eliminate the *E. faecalis* biofilm^11^; however, they may be toxic, especially for stem cells, and may result in cell death.^12,13^ Furthermore, the use of TAP results in coronal discoloration mainly because of its minocycline content.^14^

 Simvastatin is derived from lovastatin and is a semisynthetic medication. Its side chain has a single methyl group. It inhibits hydroxymethyl glutaryl coenzyme reductase that prevents endogenous cholesterol synthesis.^15^ A previous study showed that simvastatin treatment promoted odontogenic differentiation of DPSCs and mineralized tissue formation.^16^ Also, some others indicated that simvastatin prevented bacterial proliferation and biofilm formation and decreased the synthesis of extracellular polymeric substances.^17-19^

 Considering controversies regarding the efficacy of different concentrations of TAP and the gap of information regarding the antibacterial effect of simvastatin on *E. faecalis* when applied as a medicament, this study aimed to compare the antibacterial effects of TAP (ciprofloxacin, metronidazole, and doxycycline) and different concentrations of simvastatin (1‒10 mg/mL) on 4- and 6-week *E. faecalis* biofilms.

## Methods

###  Preparation of the specimens

 Seventy single-rooted mature premolars, extracted as part of the orthodontic treatment plan or due to periodontal disease, were used in this in vitro study. The teeth had no root caries, external or internal root resorption, cracks, fractures, or previous root fillings. The attached hard and soft tissues were eliminated using an ultrasonic scaler (Cavitron, Dentsply Ltd., Wex Bridge, UK), and the samples were kept in 0.5% chloramine T solution until the study tests began. The crowns were cut to yield a standard 12-mm root length. Working length was determined by a #15 K-file (Dentsply, Maillefer, Ballaigues, Switzerland). Then root canal shaping and preparation procedures were completed with the ProTaper system (Dentsply, Maillefer, Ballaigues, Switzerland) to F3 as the final apical size. Irrigation with 2.5% NaOCl was performed in the instrumentation process, followed by irrigation with 1 mL of 5.25% NaOCl for 3 minutes and 1 mL of 17% EDTA (Aria dent, Asia Chemi Teb, Tehran, Iran) for 3 minutes to remove the smear layer. Sterile saline was used for the final rinse. The outer surfaces and root apices were coated with nail varnish and resin to prevent bacterial leakage.

 The specimens underwent autoclave-sterilization at 121ºC and 15 psi for 20 minutes and stored in brain-heart infusion (BHI) broth (Merck, Germany) for 24 hours at 37ºC.

###  Biofilm formation

*Enterococcus faecalis* (ATCC 29212) was incubated in a blood agar medium at 37ºC in a humid environment with 10% CO_2_ for 24 hours. To prepare a bacterial suspension, the grown bacterial colonies were uniformly dissolved in BHI broth to obtain 0.5 McFarland standard turbidity corresponding to 1.5×10^8^ cells/mL.

**Figure 1:**
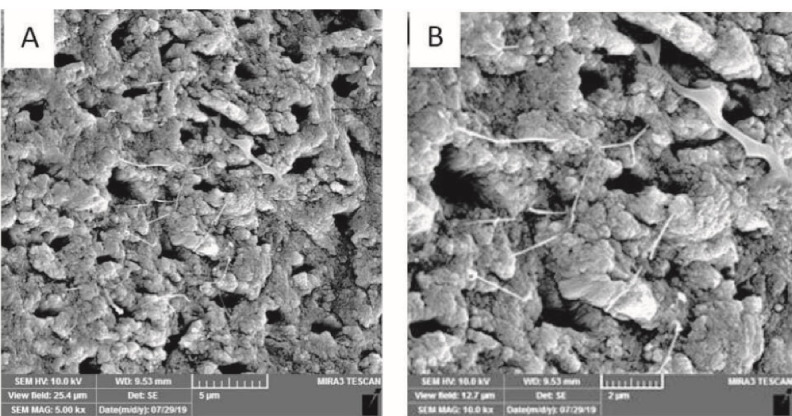
Scanning electron microscopic micrograph showing the 4-week biofilm formed on the root dentin surface (A. ×5000 – B. ×10000 magnification).

**Figure 2:**
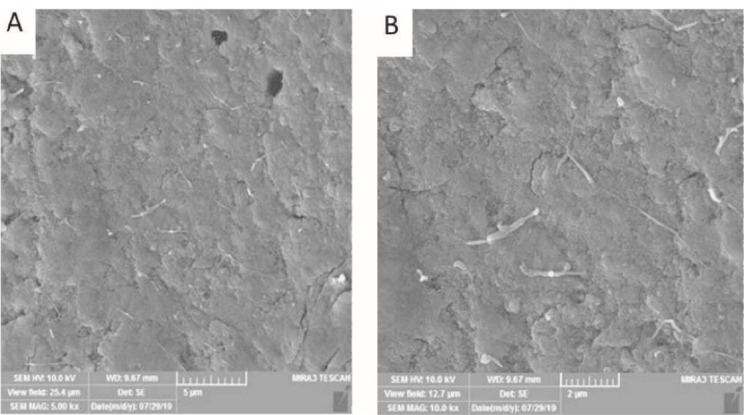
Scanning electron microscopic micrograph showing the 6-week biofilm formed on the root dentin surface (A. ×5000 – B. ×10000 magnification).

 Next, 1 mL of the bacterial suspension was transferred into each root canal. They were then placed in sterile vials, and fresh medium was added into the canals daily. To obtain 4-week (group A) and 6-week (group B) *E. faecalis* biofilms, this procedure was repeated for 4 and 6 weeks, respectively. To ensure biofilm formation, five specimens were chosen randomly from each group after the completion of the periods mentioned above and inspected using scanning electron microscopy (Model: MRIA3-FEG-SEM Tescan, Brno, Czech) (Figures 1 and 2).

 The samples in each group were randomly assigned to five subgroups (n = 12) for use of medicaments with different concentrations: subgroup 1: 1 mg/mL TAP, subgroup 2: 10 mg/mL TAP, subgroup 3: 1 mg/mL simvastatin, subgroup 4: 10 mg/mL simvastatin, and subgroup 5: positive control (phosphate-buffered saline solution).

###  Preparation and application of medicaments

 To prepare 1- and 10-mg/mL concentrations of TAP, an equal amount of the three antibiotic powders, including ciprofloxacin (Razak, Tehran, Iran), metronidazole (Pars Darou, Tehran, Iran), and doxycycline (Razak, Tehran, Iran), were dissolved in 10 mL and 1 mL of sterile distilled water, respectively. The solution was placed on a heater stirrer for 2 hours to ensure optimal dissolution. The antibiotic paste was prepared in a paste-like consistency for easy handling and delivery into the root canal. To enhance paste formation and stabilize the medicament in the root canal, 4% methylcellulose (Merck, Germany) was added to the solution in a 4% ratio, and the mixture was placed on a heater stirrer using a magnet for 2 hours at 40‒50°C to ensure homogeneity.

 To prepare 1- and 10-mg/mL simvastatin, 10 mg of simvastatin powder was dissolved in 10 mL and 1 mL of sterile distilled water, respectively. Then 4% methylcellulose was added to obtain a gel form.

 The medicaments were transferred into the canals in an aseptic environment under a biologic hood using a #40 Lentulo. Then, sticky wax was applied to provide a coronal seal to minimize the risk of contamination. Finally, the specimens were transferred to a 48-well plate in a standing position and incubated for one week at 37°C under 98% humidity.

###  Sampling 

 After one week, the samples were retrieved from the 48-well plate, the coronal wax sealing of the specimens was removed by a sterile spatula, and the medicaments were removed using 10 mL of sterile saline solution. Next, dentinal samples were collected using a sterile F4 ProTaper rotary file. The dentinal shavings were cultured on BHI agar in 8×8 cm plates by the spreading technique and incubated for 24 hours. Next, the number of *E. faecalis* colonies was counted by a colony counter and reported as colony-forming units per milliliter (CFUs/mL).

###  Statistical analysis

 The collected data were statistically analyzed using SPSS 2017. The subgroups were compared regarding the colony counts by the Kruskal-Wallis, Mann-Whitney U, and Wilcoxon tests (α = 0.05).

## Results

Table 1 presents the median and the first and third quartiles of the studied groups and their comparison results. According to this table, the median of bacterial counts in 4-week biofilms in the control group and 1-mg/mL and 10-mg/mL simvastatin groups were 400 (200‒600), 600 (320‒1600), and 80 (30‒140) units, respectively. Based on the results of the Kruskal-Wallis test, the differences in the medians of these three groups were statistically significant (*P* = 0.001). In the 4-week biofilms, the median of bacterial counts in the control group and 1-mg/mL and 10-mg/mL TAP groups were 400 (200‒600), 285 (142.5–540), and 10 (0–40) units, respectively. Based on the results of the Kruskal-Wallis test, the differences between the medians of these three groups were statistically significant (*P* = 0.001).

**Table 1:** Colony counts in the study groups

**Group**	Min*	Max*	Median* (Q1-Q3)	***P*** ** value**
Control (4 wk)	200	700	400 (200‒600)	0.001
Simvastatin 1 mg/mL (4 wk)	320	1600	600 (320‒1600)	
Simvastatin 10 mg/mL (4 wk)	0	300	80 (30‒140)	
Control (4 wk)	200	700	400 (200‒600)	0.001
TAP 1 mg/mL (4 wk)	85	102	285 (142.5–540)	
TAP 10 mg/mL (4 wk)	8	2	10 (0–40)	
Simvastatin 1 mg/mL (4 wk)	320	1600	600 (320‒1600)	0.073
TAP 1 mg/mL (4 wk)	85	102	285 (142.5–540)	
Simvastatin 10 mg/mL (4 wk)	0	300	80 (30‒140)	0.038
TAP 10 mg/mL (4 wk)	8	2	10 (0–40)	
Control (6 wk)	200	700	400 (200‒600)	0.494
Simvastatin 1 mg/mL (6 wk)	10	580	350 (250‒500)	
Simvastatin 10 mg/mL (6 wk)	10	800	160 (40‒600)	
Control (6 wk)	200	700	400 (200‒600)	0.005
TAP 1 mg/mL (6 wk)	0	230	15 (0‒125)	
Simvastatin 1 mg/mL (6 wk)	10	580	350 (250‒500)	0.008
TAP 1 mg/mL (6 wk)	0	230	15 (0‒125)	
Simvastatin 1 mg/mL (4 wk)	320	1600	600 (320‒1600)	0.165
Simvastatin 1 mg/mL (6 wk)	10	580	350 (250‒500)	
Simvastatin 10 mg/mL (4 wk)	0	300	80 (30‒140)	0.038
Simvastatin 10 mg/mL (6 wk)	10	800	160 (10‒600)	
TAP 1 mg/mL (4 wk)	60	600	285 (143‒540)	0.534
TAP 1 mg/mL (6 wk)	0	230	15 (0‒125)	

*CFU/mL

 A comparison of bacterial counts obtained from the statistical analyses of the TAP and simvastatin group in the 4-week biofilms showed no statistically significant difference between the medians of simvastatin and TAP groups in the 1-mg/mL concentration (*P* = 0.073). In contrast, there was a significant difference between the two groups with the 10-mg/mL concentration of TAP (*P* = 0.038). The medians of bacterial counts in the groups of 10-mg/mL simvastatin and 10-mg/mL TAP were 80 (30‒140) and 10 (0‒40), respectively.

 In the 6-week biofilms, the differences between the medians of simvastatin groups with the 1-mg/mL and 10-mg/mL concentrations and the control group were not statistically significant (*P* = 0.49). Based on the Mann-Whitney U test, this index was significant, given the difference between the medians of the bacterial counts in the 1-mg/mL concentration of TAP and control groups in the 6-week biofilms (*P* = 0.005). Likewise, based on the Mann-Whitney U test, there was a significant difference between the medians of bacterial counts with the 1-mg/mL concentrations of simvastatin and TAP (*P* = 0.005).

 The Wilcoxon test was conducted to compare the bacterial counts in the studied groups between the 4- and 6-week biofilms. Accordingly, the only statistically significant difference was observed in the medians of bacterial counts in the 10-mg/mL simvastatin group (*P* = 0.038). The median of the first and third quartiles for 10-mg/mL simvastatin in the 4- and 6-week biofilms were 80 (30‒140) and 160 (10‒600), respectively.

## Discussion

 Vital and necrotic pulp tissue residues can be removed by chemomechanical disinfection of root canals, and the microbial biofilm can be disrupted. Thus, chemomechanical preparation may significantly enhance the success of root canal therapy.^20^ Isolation of *E. faecalis* has been reported from secondary and persistent infections. It can be easily cultured and forms a calcified monoculture biofilm after three weeks.^21^ Since this microorganism possesses some virulence and resistance mechanisms to antimicrobial agents by forming a biofilm, it is commonly used as the reference microorganism for research purposes.^22^ TAP and simvastatin serve the purpose of removing potentially pathogenic microorganisms.^23^ The literature has reported the revascularization of immature necrotic molars by TAP.^24^

 Unlike most antimicrobial studies, this study focused on biofilm rather than the planktonic form of bacteria, which is more resistant than the planktonic form.^25^ The present study used extracted teeth to better simulate the clinical setting. After culture and biofilm formation in the root canals, antimicrobial agents were applied to the canals for one week to assess the inhibitory effect of dentin on the antimicrobial activity of the applied agents.^26^ Also, the number of colonies was counted to assess the antibacterial activity, which is more accurate than the agar diffusion test used in previous studies.^27-29^

 An in vitro study showed the interactions of *E. faecalis *with the root dentin after four weeks of incubation. A mature biofilm structure with crystals and calcifications adjacent to the dentin-biofilm interface was noted after six weeks of incubation.^30^ This finding was in line with the present results showing that TAP and simvastatin at 10 mg/mL concentration were more effective against the 4-week biofilms than the 6-week biofilms.

 Reyhani et al^11^ showed that 1000, 100, and 10 mg/mL concentrations of TAP perfectly eliminated *E. faecalis*. In contrast, TAP at 1, 0.1, and 0.01 mg/mL concentrations significantly decreased the mean colony counts in comparison with the control group. Similar findings were reported in the present study with 1‒10 mg/mL TAP against the 6-week biofilms such that the difference between the control group and 1 mg/mL TAP was statistically significant (*P* = 0.005). Furthermore, in using TAP with 10 mg/mL concentration against the 6-week biofilms, only one specimen showed bacterial growth.

 Sabrah et al^31^ showed that TAP significantly decreased the bacterial count but was toxic and significantly decreased the viability of DPSCs except for the lowest (0.25 and 0.125 mg/mL) concentrations that were not toxic for DPSCs. The antibacterial effect of TAP reported in their study was consistent with the present results. Considering the results, TAP at 1‒10 mg/mL concentrations used in the present study may be toxic for DPSCs.

 Also, Chuensombat et al^13^ indicated that 25.00 ug/mL TAP perfectly eradicated the bacteria; however, they reported an increase in cytotoxicity in a concentration- and time-dependent manner. The effective concentrations of TAP were different in the current study since *E. faecalis* biofilms were evaluated.

 In the current study, the selected concentrations of simvastatin matched those of TAP. Graziano et al^32^ showed that simvastatin at 62.5 ug/mL concentration inhibited biofilm formation and adhesion of *Staphylococcus aureus*. Also, simvastatin decreased cell viability and was effective against mature biofilms. The latter study showed a positive effect of TAP and simvastatin, consistent with the present study. However, due to the wide range of available microorganisms, different effects of antimicrobial agents, and limitations of the available studies, further investigations are necessary to obtain more accurate results.

 Studies have shown that simvastatin stimulates DPSCs both in vitro and in vivo and induces pulp tissue regeneration following pulpotomy,^16,33,34^ which might have had a positive effect on DPSCs in regenerative procedures as an antimicrobial agent.

 One of the limitations of this study is comparing the antibacterial effect of simvastatin with high concentrations of TAP (1 and 10 mg/mL). According to the AAE guideline,^35^ these concentrations for TAP have a cytotoxic effect on stem cells.

## Conclusion

 Considering the present in vitro results, the antibacterial activity of 10 mg/mL concentration of TAP and simvastatin was more than that of 1 mg/mL concentration against the 4- and 6-week *E. faecalis* biofilms. Furthermore, TAP and simvastatin with 10 mg/mL concentration were more effective against the 4-week *E. faecalis* biofilms than the 6-week biofilms. According to the present results and since biofilms may remain viable in the root canal system for weeks to months, the application of TAP and simvastatin with 10 mg/mL concentration appears to be more effective, although further studies are required.

## Author Contributions

 SR: Manuscript review and editing; NG: Study concept and design, study procedures; PD: Manuscript preparation and editing; IT: Study procedures; MF: Manuscript preparation and edit; NRD: Manuscript editing; EK: Manuscript editing.

## Funding

 None.

## Ethics Approval

 This study was approved by the Ethics Committee of Tabriz University of Medical Sciences (IR.TBZMED.REC.1398.378).

## Competing Interests

 The authors declare no competing interests with regard to the authorship and/or publication of this article.
